# Supplementary material for: Chemical crosslinking extends and complements UV crosslinking in analysis of RNA/DNA nucleic acid–protein interaction sites by mass spectrometry
Source: Nucleic Acids Res. 2025 Aug 18;53(15):gkaf727. doi: 10.1093/nar/gkaf727 (PMC12359043; doi:10.1093/nar/gkaf727)

Supplementary File S2. Exemplary mass spectra of UV-induced E. coli nucleotide-peptide crosslinks (S30 fraction, 2 replicate) identified by NuXL, but not by MSFragger-Labile workflow, visualized by OpenMS ToppView from NuXL output

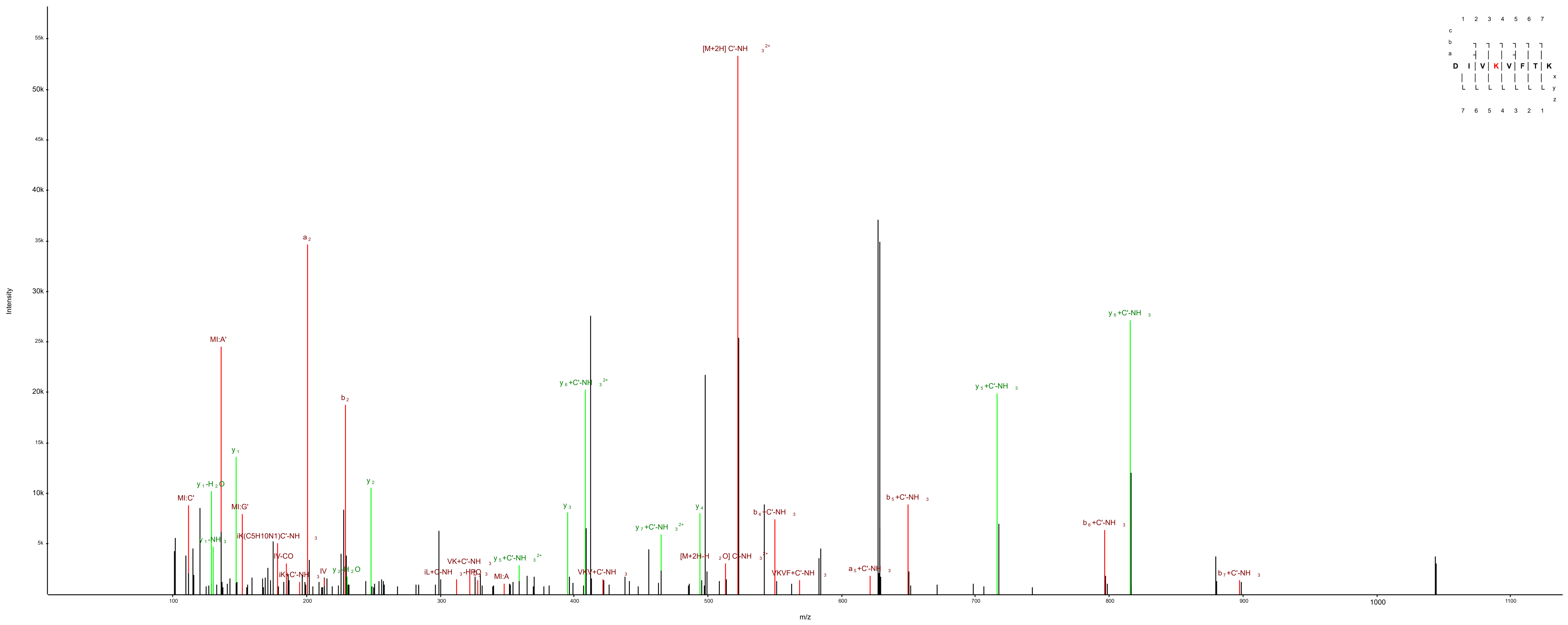

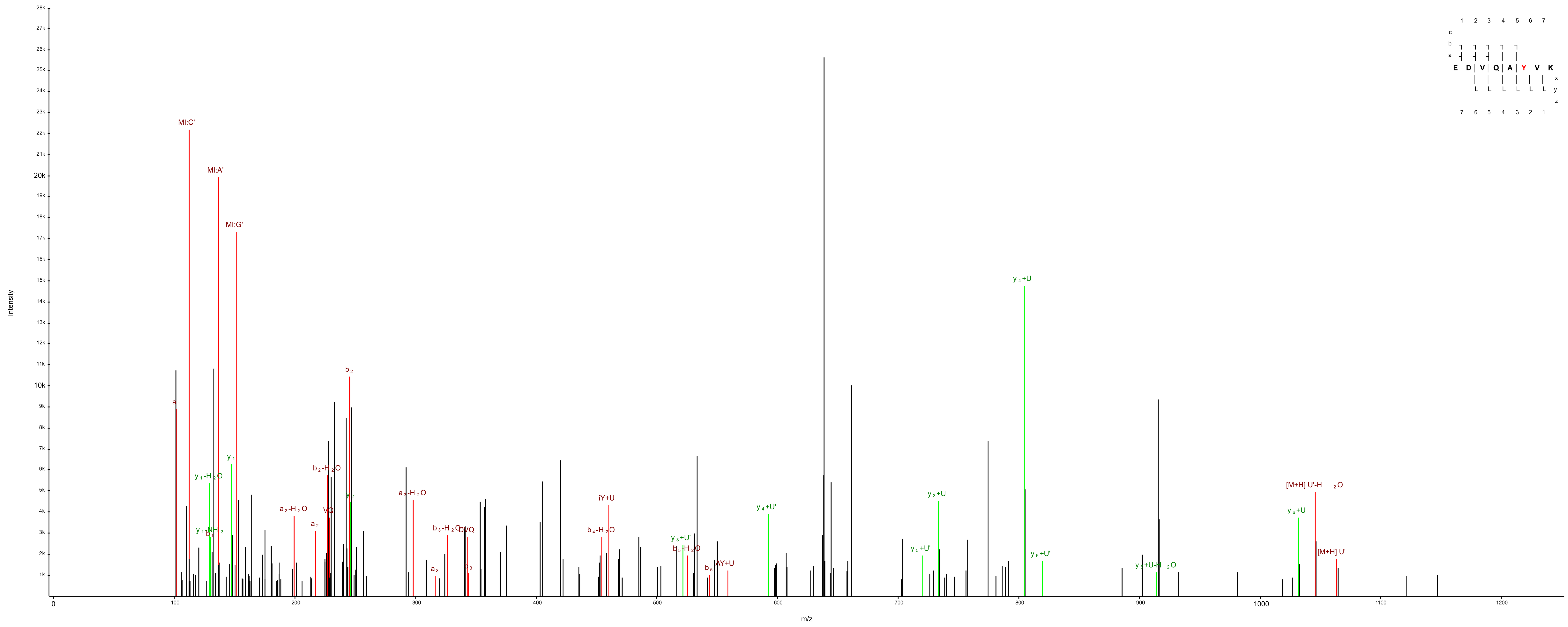

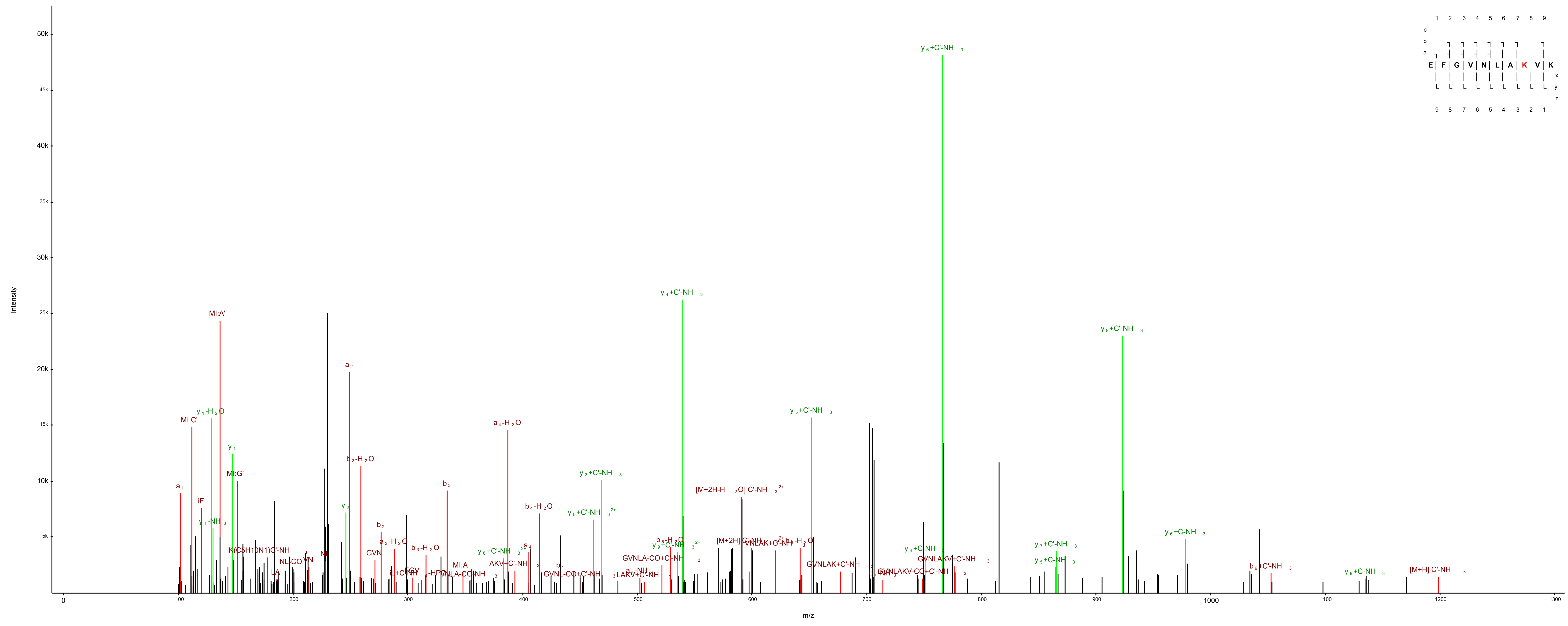

Supplement: gkaf727_Supplemental_Files [file gkaf727_supplemental_files.zip › SupplementaryFileS3.pdf]
