# Supplementary material for: Chemical crosslinking extends and complements UV crosslinking in analysis of RNA/DNA nucleic acid–protein interaction sites by mass spectrometry
Source: Nucleic Acids Res. 2025 Aug 18;53(15):gkaf727. doi: 10.1093/nar/gkaf727 (PMC12359043; doi:10.1093/nar/gkaf727)

Supp. Fig. S2A

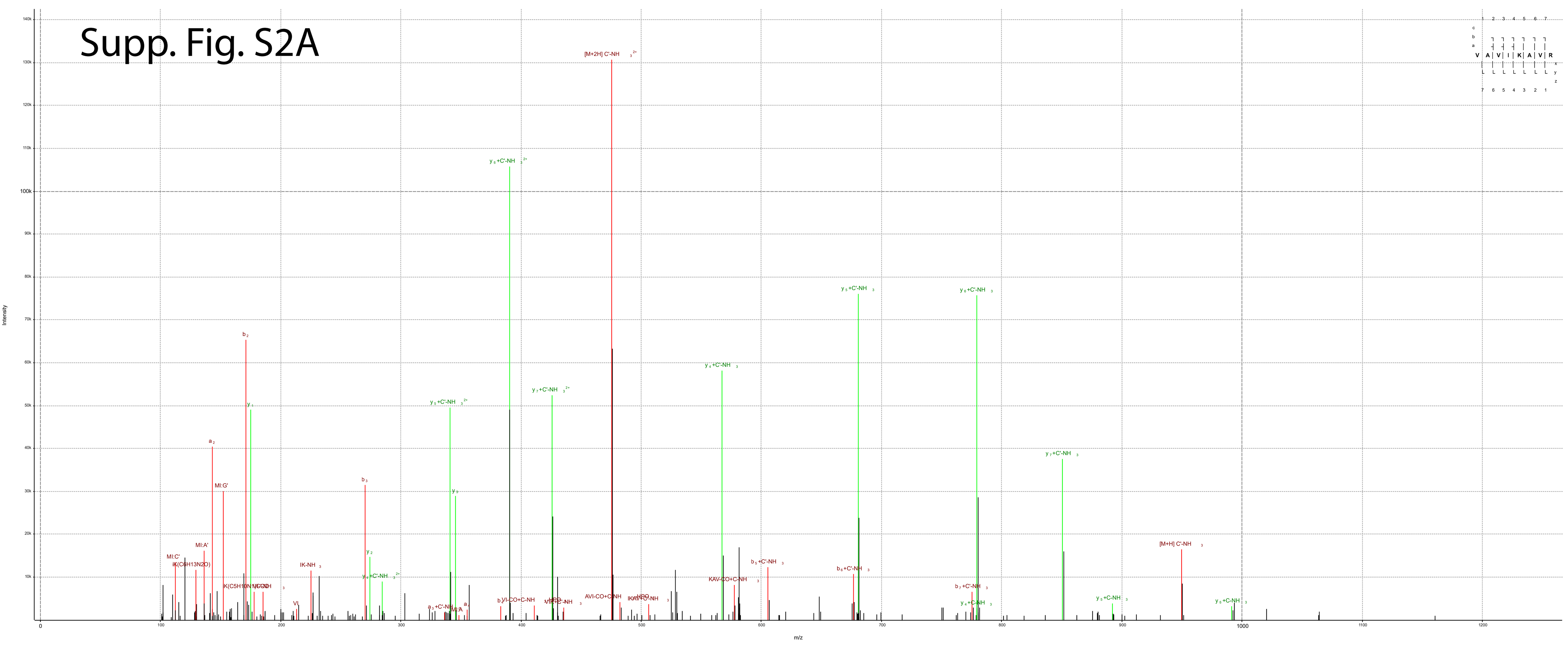

Supp. Fig. S2B

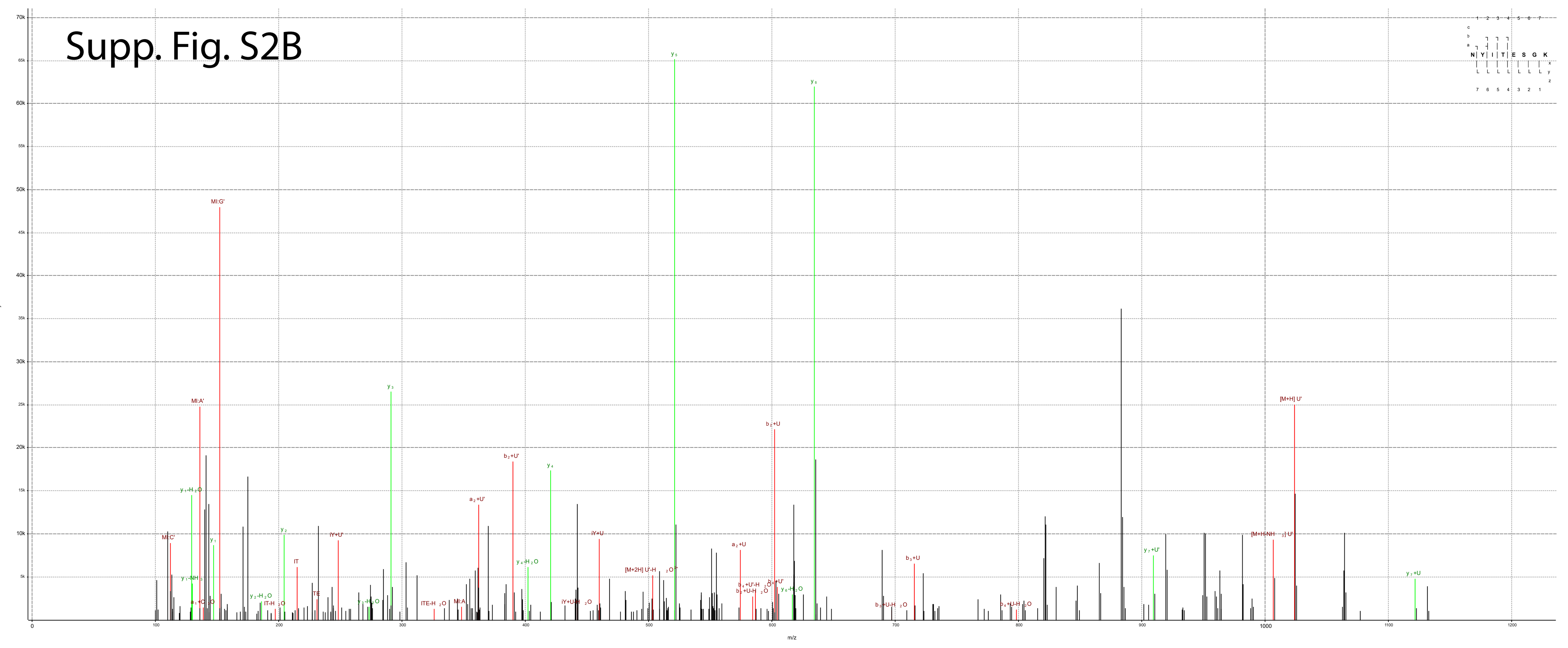

Supp. Fig. S2C

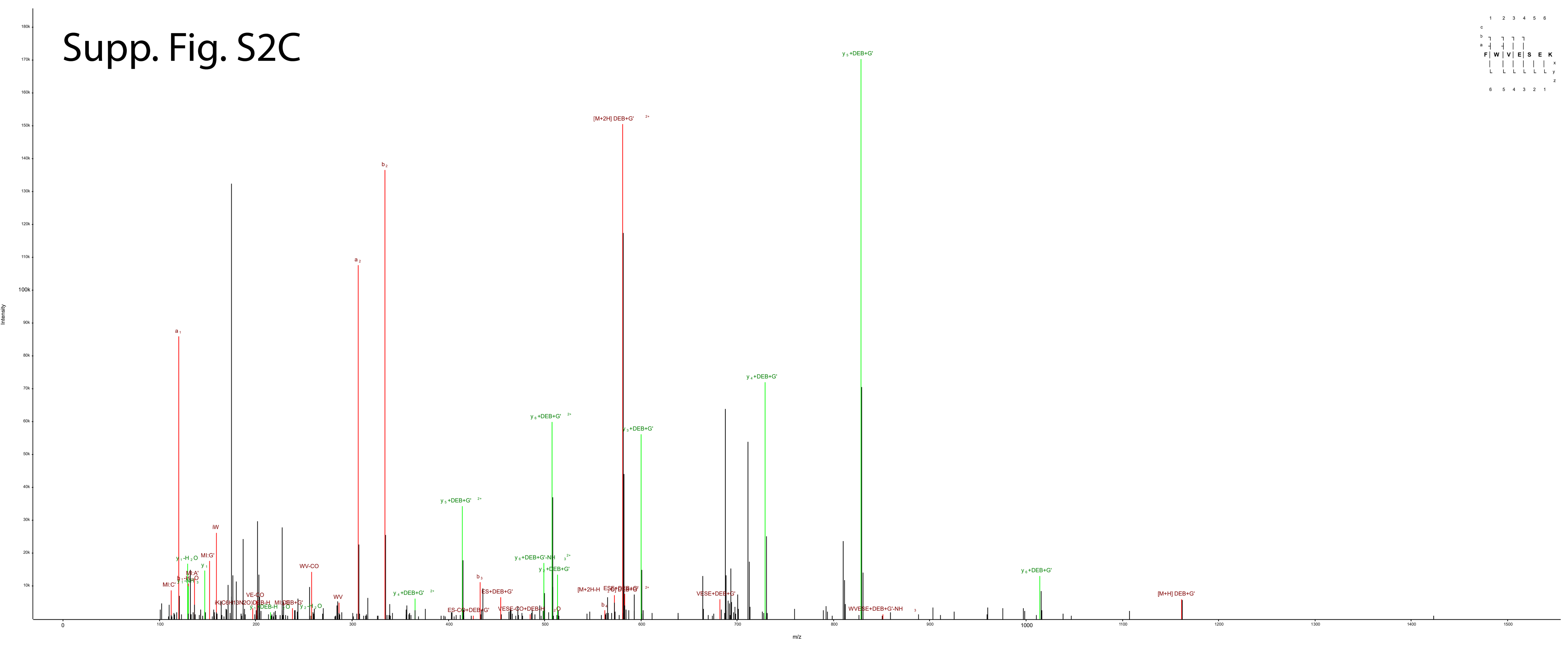

Supp. Fig. S2D

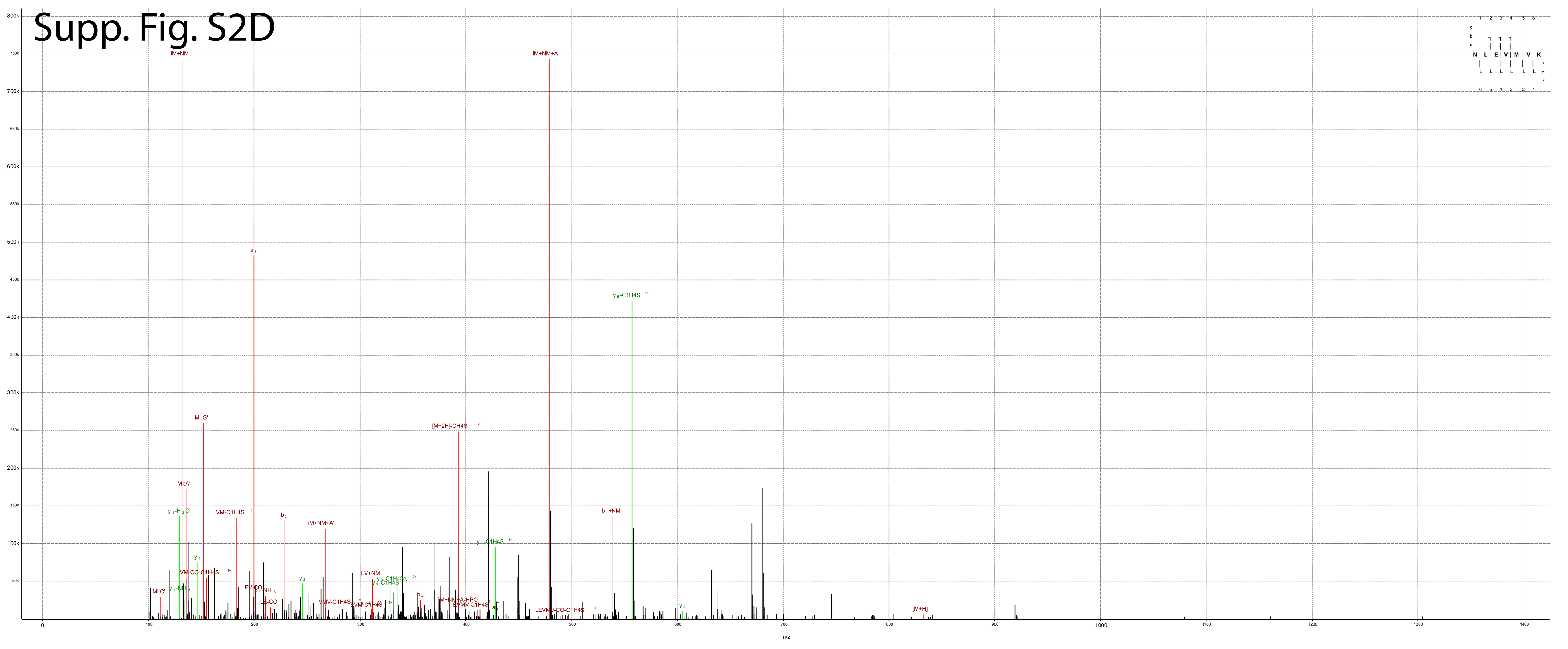

Supp. Fig. S2E

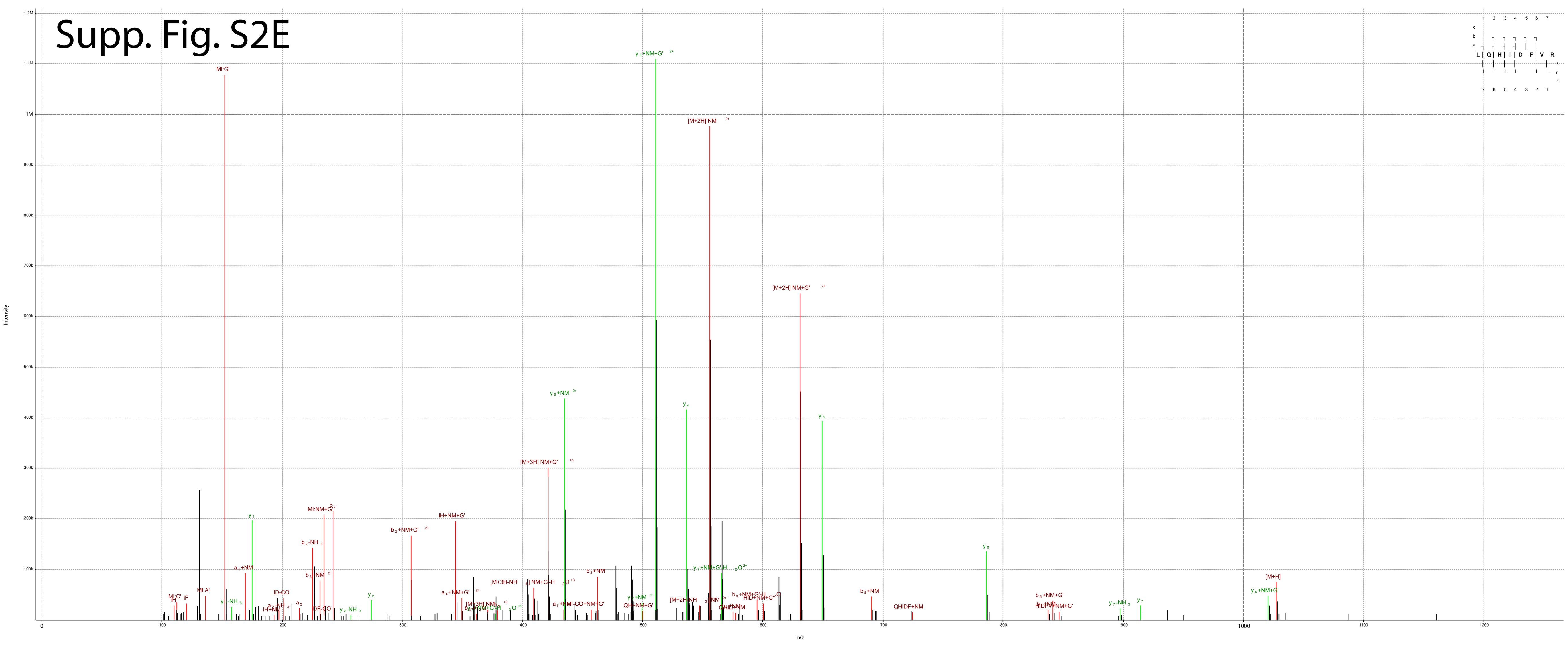

Supp. Fig. S2F

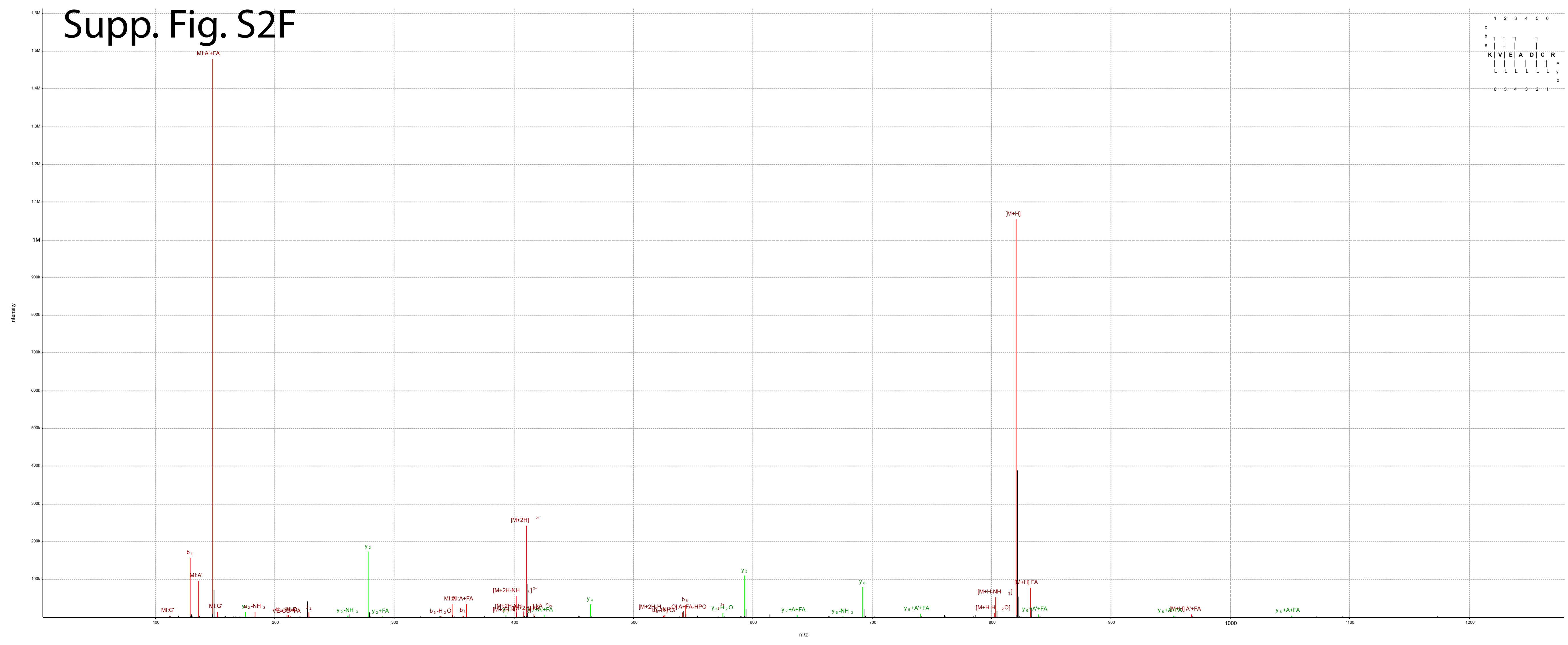

Supplement: gkaf727_Supplemental_Files [file SupplementaryFileS2.pdf › SupplementaryFileS2.pdf]
